# Inversion of the Uterus

**Published:** 1902-10-25

**Authors:** 


### INVERSION OF THE UTERUS.

At the last meeting of the Obstetrical Society Mr. J. W. Taylor gave details of a case of complete inversion of the uterus of seven months' duration which had caused dangerous hæmorrhage and obstinate vomiting. In the treatment of this case attempts to effect reduction by means of elastic pressure with repositors having failed, he had in the end to perform anterior vaginal cœliotomy, anterior uterotomy, and replacement. The line of treatment adopted led to considerable discussion, and although Mr. Taylor was congratulated upon his success there seemed to be a general concensus of doubt as to the desirability or necessity of often adopting such measures as he had described. Dr. Handfield Jones regretted that he could not agree with the line of treatment adopted; he had had a case of complete inversion in which the same difficulty had arisen as in Mr. Taylor's case—viz, that the womb was so movable that it was extremely difficult to keep the cup of the Aveling's repositor on the uterine body. But by packing swabs round the womb in the upper vagina, and by patient watching, reposition had at length been secured. Professor Herbert Spencer spoke of the value of Aveling's repositor in such cases. In his opinion there was no method of reducing an inverted uterus to compare with reposition with this instrument and the cases must be very rare in which it would fail. The difficulty caused by slipping of the cup off the fundus often occurred, but by care and patience this could be overcome. Dr. W. Duncan also thought that if Aveling's repositor had been applied to the inversion, and kept there by packing the vagina carefully all round, the inversion would have been reduced, and he mentioned a case in which by this method he had reduced a complete inversion of the uterus which had existed for nine years. Dr. G. F. Blacker pointed out that the question of a future pregnancy had to be considered. In a recent case of his own, in which he had reduced a chronic inversion of four months' standing by Aveling's repositor, the patient became pregnant and had lately been delivered of a full-term child, and as these cases usually occurred in young women who might subsequently become pregnant it was necessary to bear in mind the danger that would exist under such circumstances of rupture of the uterus as a consequence of the uterotomy, especially where, as in Mr. Taylor's case, it was necessary to carry the incision from the fundus uteri to the external os.

